# A Cross-Talk between Diet and the Oral Microbiome: Balance of Nutrition on Inflammation and Immune System’s Response during Periodontitis

**DOI:** 10.3390/nu14122426

**Published:** 2022-06-11

**Authors:** Simona Santonocito, Amerigo Giudice, Alessandro Polizzi, Giuseppe Troiano, Emanuele Maria Merlo, Rossana Sclafani, Giuseppe Grosso, Gaetano Isola

**Affiliations:** 1 Unit of Periodontology, Department of General Surgery and Surgical-Medical Specialties, School of Dentistry, University of Catania, 95124 Catania, Italy; simonasantonocito.93@gmail.com (S.S.); sclafanirossana@yahoo.it (R.S.); gaetano.isola@unict.it (G.I.); 2 Unit of Dentistry, Department of Health Sciences, University of Catanzaro “Magna Graecia”, 88100 Catanzaro, Italy; a.giudice@unicz.it; 3 Department of Clinical and Experimental Medicine, University of Foggia, 71122 Foggia, Italy; giuseppe.troiano@unifg.it; 4 Department of Human and Pediatric Pathology “Gaetano Barresi”, University of Messina, 98122 Messina, Italy; emanuelemaria.merlo@unime.it; 5 Department of Biomedical and Biotechnological Sciences, University of Catania, 95123 Catania, Italy

**Keywords:** nutrients, periodontitis, oral health, diet, macronutrients, micronutrients, microbiome, oral dysbiosis, immune response

## Abstract

Over the last few decades, studies on the oral microbiome have increased awareness that the balance between the host and the microbial species that coexist in it is essential for oral health at all stages of life. However, this balance is extremely difficult to maintain, and many factors can disrupt it: general eating habits, sugar consumption, tobacco smoking, oral hygiene, and use of antibiotics and other antimicrobials. It is now known that alterations in the oral microbiota are responsible for developing and promoting many oral diseases, including periodontal disease. In this context, diet is an area for further investigation as it has been observed that the intake of particular foods, such as farmed animal meat, dairy products, refined vegetable oils, and processed cereals, affects the composition of the microbiota, leading to an increased representation of acid-producing and acid-tolerant organisms and periodontal pathogens. However, little is known about the influence of diet on the oral microbiome and the creation of a suitable microenvironment for the development of periodontal disease. The aim of the present study is to evaluate current knowledge on the role of diet in the oral dysbiosis underlying periodontal disease.

## 1. Introduction

The human oral cavity hosts the second most plentiful and diverse microbiota after the gastrointestinal tract [1,2]. Antonie van Leeuwenhoek first found and observed the existence of microbes in dental plaque under a microscope. Dental plaque has been studied qualitatively [3] from then on. Currently, the human oral microbiome is believed to consist of more than 250 species, including several key pathogens, such as *Streptococcus mutans*, *Porphyromonas gingivalis (P. gingivalis)*, *Tannerella forsythia (T. forsythia)*, and *Aggregatibacter actinomycetemcomitans*, implicated in the etiology of periodontal disease [4]. Periodontitis is a disease with a multifactorial etiology that, following infection induced by bacteria of the periodontal biofilm, can determine tooth loss following host response [5]. During recent years, the improvements in genetic techniques allowed a better identification of bacteria involved in periodontitis, including not only data on periodontopathogenic processes but also further information on the overall oral microbial networks contributing to the pathogenesis of the disease. In this context, it is important to understand the variations in the oral microbiome composition between health and disease, both qualitatively and quantitatively [6,7].

The induction and progression of the disease are promoted by a dysbiosis of the oral commensal microbiota, which may affect the host’s immune defenses, causing an inflammatory response that ultimately leads to irredeemable destruction of the periodontium (i.e., alveolar bone and periodontal ligament) in a susceptible host [7,8,9]. Periodontal disease severity is related to both modifiable (smoking, diet) and non-modifiable host risk factors (genetic susceptibility) as well as environmental factors [10,11,12]. The reduction in pathogenic periodontal bacteria in biofilms by mechanical instrumentation [13] through nonsurgical periodontal treatment has been shown to have limited long-term benefits [14,15]. The persistence of periodontal inflammation can result in residual pockets and the persistent of periodontal bacteria, leading to new tissue destruction [16,17].

In relation to the central role played by inflammation in the initiation and evolution of periodontitis, there is a growing interest in adjuvant therapies and their action in reducing inflammatory mediators. Several nutrients have an important impact on periodontal health [18]. Evidence indicates that macronutrients and micronutrients can modulate pro-inflammatory and anti-inflammatory cascades, influencing a person’s baseline inflammatory status [19]. Diet provides nutritional sources for the oral microbiota and exerts a selective pressure, favoring the survival and replication of organisms best adapted to utilize specific food resources derived from the host [8]. The influence of diet on the oral microbiome has become more evident over the last decades while observing a shift toward a “Westernized” dietary pattern characterized by staple foods, such as farmed animal meats, high-sugary dairy products, refined vegetable oils, and processed grains that were accompanied by pathological changes in the oral microbiota, including increased representation of acid-producing and acid-tolerant organisms [3,20]. The objective of this review is to group the latest scientific evidence regarding the impact that diet may have on oral dysbiosis associated with periodontal disease.

## 2. Oral Microbiome

**Figure 1:**
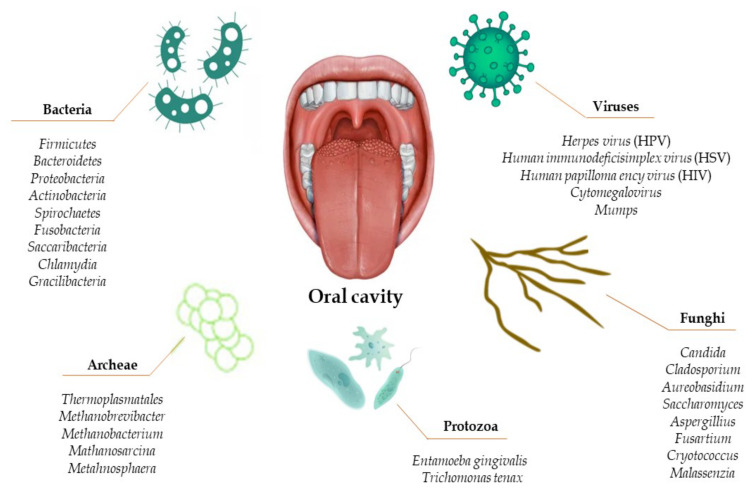
Composition of the human oral microbiota.

The oral microbiota is the second most complex microbiota in the human body, after the intestinal microbiota [4,21]. Its complexity lies in the multitude of micro-organisms hosted in the mouth, such as bacteria, fungi, viruses, archaea, and protozoa (Figure 1) [22,23,24,25,26].

It can be seen from the database of the human oral microbiota that there are approximately 700 microbial species that make up the oral microbiota, but only 54% are actually known as cultured and named, while 14% are cultured but not named, and the remaining 32% are uncultivated phylotypes [6,27].

The complexity of the oral ecosystem depends on the different characteristics of its components, including the saliva, the soft tissue surfaces of the oral mucosa and tongue, and the hard tissue surfaces of the teeth. Each component provides a unique ecosystem with optimal conditions and nutrients for the microbes that inhabit it [7,28]. In fact, it has been demonstrated that samples taken from the same site in several individuals had more similar characteristics than samples from the same individual taken from different niches [8,29].

Over the last few decades, great strides have been made in understanding the oral microbiota composition. The development of new techniques for identifying microorganisms led to an abandonment of culture-dependent techniques in favor of more sophisticated and less labor-intensive techniques [4,30]. Among the main problems associated with the culture of micro-organisms is the difficulty of recreating in vitro the conditions under which these micro-organisms live in vivo, such as specific nutrients, accurate temperature and pH, and interaction with other micro-organisms in their community [9,13]. Current techniques, which are culture-independent, include 16S ribosomal RNA sequency, which has made it possible to better estimate the oral microbiota composition [4,31]. The Human Oral Microbiome Database provides a list and detailed description of the features and genomic information of the currently known oral cavity bacteria [32,33].

The main constituents of the oral microbiota are bacteria [4,34]. The oral bacterial community consists of six main phyla—Firmicutes, Bacteroidetes, Proteobacteria, Actinobacteria, Spirochaetes, and Fusobacteria—which account for 94% of the taxa detected. The remaining phyla—*Saccharibacteria*, *Synergistetes*, *SR1, Gracilibacteria*, *Chlamydia*, *Chloroflexi*, *Tenericutes,* and *Chlorobi*—contain the remaining 6% of the taxa [32]. Most of the isolated taxa were detected in the gingival sulcus, while to a significantly lesser extent they were detected on the tongue, soft palate, hard palate, tooth surface, and tonsils. It is worth noting that although there is an overlap of species isolated in different oral habitats, some species are site specific [8]. On the other hand, the bacteria in saliva are derived from a mixture of microbes excreted from all the sites mentioned before and constitute the salivary microbiota [35]. The combination of the salivary microbiota and the organismic and inorganic components contained in saliva constitute the salivary biofilm. In detail, saliva is produced by the major salivary glands (parotid, submandibular, sublingual) and minor glands. It is characterized by a pH of about 6.7 and contains countless molecules: proteins, glycoproteins, sugars, and inorganic ions. On the one hand, they represent the primary nutrient source for the resident oral microbiota and, on the other hand, play a role in defending and protecting against insults that could disrupt the health of the oral cavity and, more generally, the entire body. As soon as the surface of a tooth erupts or is cleaned, salivary proteins and glycoproteins are adsorbed, forming a film on the surface of the teeth on which bacteria can adhere. This film provides a favorable substrate for bacterial growth and survival and defends against external insults such as diet, drug intake, etc. [36].

Contrary to popular belief, fungi are an integral part of the healthy oral microbiota. Only under certain conditions do they become opportunistic pathogens, such as disruption of the microbiota balance or in immunocompromised subjects [10]. It has been observed that the microbiota of healthy individuals can present up to 101 species of fungi. Candida species are the most common, followed by *Cladosporium*, *Aureobasidium*, *Saccharomyces*, *Aspergillus*, *Fusarium,* and *Cryptococcus* [11,34].

Archaea constitutes only a minor part of the oral microbiome, and the species observed are all methanogens: *Thermoplasmatales*, *Methanobrevibacter*, *Methanobacterium*, *Methanosarcina*, and Methanosphaera [12]. They were found with a higher prevalence in individuals with periodontitis [13].

Saliva and soft tissue appear to have a similar microbial biofilm [14]. Approximately 3621 bacterial taxa from 98 healthy subjects were identified in saliva, with *Firmicutes* (genus *Streptococcus* and *Veillonella*) and Bacteroidetes (genus *Prevotella*) being the predominant phyla [15]. It is believed that the proteins present may promote microbial adhesion to oral mucosal surfaces and teeth, as well as agglutination and removal of microorganisms by swallowing saliva [14,30]. The oral mucosa has a low number of bacteria organized to form a monolayer at the level of the cheeks and palate due to rapid tissue turnover [37]. In contrast, the surface of the tongue is characterized by multilayers of bacteria arranged to form a biofilm. The predominant microbiota on the lingual dorsum of healthy subjects is reported to be *Streptococcus salivarius*, *Rothia mucilaginosa*, and an uncharacterized species of *Eubacterium* (strain FTB41) [38].

The oral microbial ecosystem is constantly exposed to exogenous foreign substances, which are key determinants of colonization and microbe resistance making up the oral microbiota [39]. Among the first colonizers of the oral cavity are *Streptococcus mitis*, *Streptococcus sanguinis*, *Streptococcus gordonii*, and *Streptococcus salivarius*, which have characteristics that make them suitable for selective colonization of the tongue mucosa and the gingival mucosa, even before the dental elements erupt [40].

**Figure 2:**
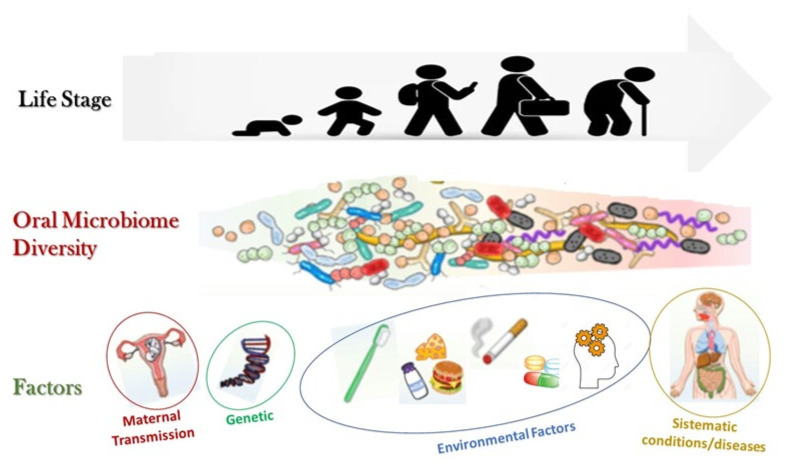
Evolution of oral microbiome.

At the moment of their eruption in the oral cavity, teeth acquire a glycoprotein protective coating, which initiates successional microbial colonization, leading to the development of complex polymicrobial biofilm communities, i.e., dental plaque (Figure 2) [17,18].

This is a structurally and functionally organized biofilm arranged on the surface of teeth, which is formed through a series of ordered stages and remains stable over time [21]. Therefore, the surface of teeth has special characteristics that favor bacterial adhesion and the formation of biofilm, i.e., bacterial plaque [14]. Depending on its location, dental plaque is differentiated into supragingival plaque and subgingival plaque [15,41]. One study observed that the supragingival plaque of healthy subjects is mainly made up of Firmicutes and Actinobacteria (genus Corynebacterium and Actinomyces), which tend to generate acid, can survive in low pH environments, and are responsible for caries [14]. As it moves from the surface of the teeth to the subgingival area, along the root, the biofilm changes its characteristics: there is more serum and less saliva, the environment becomes more anaerobic, and the pH and temperature become extreme [42]. For this reason, the composition of the subgingival biofilm is different from the supragingival biofilm and consists of 347 species falling into 9 bacterial phyla, including Obsidian Pool OP11, TM7, Deferribacteres, Spirochaetes, Fusobacteria, Actinobacteria, Firmicutes, Proteobacteria and Bacteroidetes [42].

## 3. Geographic Tongue, Psoriasis, and Oral Microbiome

Geographical tongue (GT) is a lesion of the oral mucosa affecting the dorsal and lateral surfaces of the tongue with unknown etiology. Clinically it manifests as erythematous areas, associated with papillary atrophy, that are surrounded by a whitish peripheral area. A distinctive feature is the migration of lesions to create a constantly changing map-like pattern [43]. Anatomical changes in the tongue may induce dysbiosis of the lingual microbiota, which in turn could trigger inflammation and an increase in several proinflammatory cytokines. However, other evidence would suggest that dysbiosis rather than the consequence of GT may be the cause [44]. Recently, one study observed that more bacterial taxa were identified at sites of GT injury than at healthy GT sites. However, the greater richness of bacteria at the lesion sites does not imply greater bacterial density. The phylum most prevalent at lesion sites include Firmicutes (predominant on the tongue surface) and spirochetes (associated with multiple oral infections). Fusobacteria were found significantly fewer in number. They play a role in the induction of human β-defensin-2, which has antimicrobial activities and is associated with inflammation. Changes in microbial diversity have been shown to underlie the dramatic and rapid increases in chronic inflammatory and autoimmune diseases observed in high-income countries [45]. Therefore, it is not surprising that GT may be associated with several chronic inflammatory conditions, including psoriasis. Psoriasis is an immune-mediated inflammatory disease with unknown etiology, which may be associated with defective proliferation and differentiation of keratinocytes, inducing skin and eye lesions [46]. The association between geographic tongue and psoriasis has been demonstrated in several studies, based on the observation of its core lesions, the microscopic similarity between the two conditions, and the presence of a common genetic marker, human leukocyte antigen (HLA) HLA-C*06 [47]. Recently, psoriasis has been associated with changes in the skin and gut microbiome, which may trigger psoriasis and influence the course of the disease [48]. Therefore, dysbiosis could be the link between the two diseases and inflammation, both systemic and local.

## 4. Impact of Food Texture and Diet on the Oral Microbiome

The actual influence of diet on oral microbiota has been long debated [49]. The rationale for this hypothesis relies on historical observation of two key periods in human history: the Neolithic and Industrial revolutions. These periods represented two critical junctures in our history with repercussions beyond economic, cultural, and social matters. Indeed, the foods and processing techniques that arose in these two historical periods significantly altered the central constituents of the human diet, including macronutrient proportions, glycemic load, fatty acid composition, sodium and potassium levels, micronutrient levels, dietary pH, and fiber content [41,50]. The advent of agriculture about 10,000 years ago, during the transition from the Mesolithic to the Neolithic, drastically changed the composition of the diet [51]. The pre-agricultural diet consisted mainly of unprocessed wild fruits and vegetables, tubers, nuts and wild animals [52]. The transition from hunter-gatherer to agrarian lifestyles introduced cereals, pulses, dairy products and meat into the human diet [53]. This change was associated with the appearance of dental and periodontal diseases as a result of changes in the oral microbiota [54].

**Figure 3:**
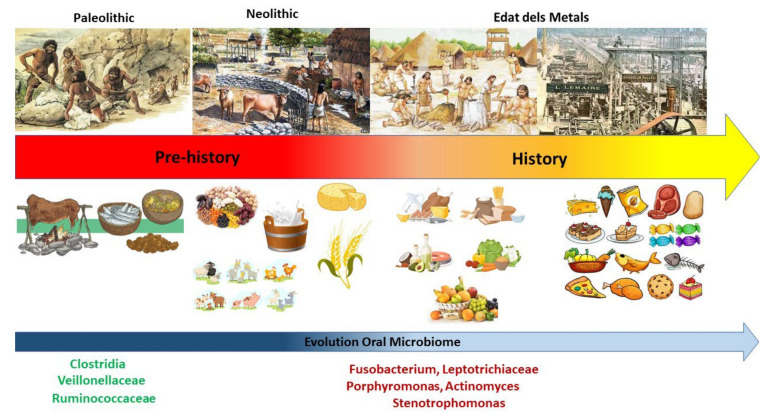
Evolution of diet and corresponding microbiome.

Little evidence of dental and periodontal disease exists for pre-neolithic hunter-gatherer societies. Changes in the oral microbiota during the Mesolithic−Neolithic transition can be observed in preserved dental calculus samples from the Mesolithic period, indicating a distinct microbial profile from dental calculus samples collected in the Neolithic, Bronze Age, Medieval and modern times. It has been observed that the microbial profiles of plaque samples from the Neolithic period are similar in terms of proportions and species of microbes present, despite dietary changes over time [37]. Compared to the proportions and species of oral microbes found in hunter-gatherer plaque samples, samples from agricultural populations from the Neolithic onwards are predominated by caries-associated species, such as those of the Veillonellaceae family, as well as taxa associated with periodontal disease, including *P. gingivalis*, *T. forsythia*, and *T. denticola* [37] (Figure 3).

An oral health study analyzed the impact of ancestral diets among “bush dwellers” and the increasingly externalized diets of “villagers” on oral health status. The results validated the hypothesis that agricultural societies show worse dental health than hunter-gatherer societies: women living in a village and consuming a predominantly agricultural diet had a significantly higher incidence of caried lesions and periodontal disease compared to those living in a bush environment and eating a diet of wild foods, including pulses, game, berries and uncultivated tubers. Men who lived in the bush did not maintain the similar oral health status of their female counterparts, presumably due to cultural influences, such as high honey ingestion and tobacco consumption, among others [55]. Recently, Wright et al. conducted a cross-sectional study on the association between diet and periodontitis among 10,000 NHANES participants. That study found that a dietary pattern rich in fruits, vegetables, salad, water, and tea and with limited intake of fermentable carbohydrates, fatty acids, protein, and sugar-rich beverages had a lower extent of periodontal disease. This is attributable to reduced expression of periodontal bacteria in the oral microenvironment [56].

Changes in the pre-neolithic oral microbiota were further exacerbated by the evolution of food manufacturing techniques as a result of industrial revolution about 200 years ago [57]. The invention of mechanical mills allowed for the refining of carbohydrates, changing the nutritional properties of cereals, which were significantly impaired by the isolation of starch-rich constituents (flour) and the elimination of the outer bran layer [58]. The processing of cereals has influenced different added features of the diet, such as total nutrient content, starch bioavailability, and texture. Therefore, the transition from hunter-gatherer to agrarian lifestyles introduced dairy products, refined carbohydrates, vegetable oils, and alcohol into the human diet, which are staples of the modern diet [53]. The introduction of these foods into the daily human routine has been linked with a decline in oral health and several other diseases, including (but not limited to) cardiovascular disease, inflammatory bowel disease, rheumatic diseases, many cancers, and obesity [55].

Several animal studies have shown the relationship between oral health and the texture of food eaten: wild animals have a higher oral health status because domestic animals eat refined foods that do not require intense chewing effort [59]. The chewing stimulates salivary flow, and that food passes through the mouth for only brief moments during the course of a day. Therefore, the main sources of nutrition for oral micro-organisms come from saliva and crevicular fluid [49]. Oral bacteria find their nutrient sources in these body fluids and work to break down the substrates they contain to obtain nutrition sources [37]. Streptococci, in particular, play a major role in obtaining sugar residues from glycoproteins [60]. Recent human studies have shown no difference in the growth rates of oral bacteria in the presence or absence of food, indicating no relationship between diet and the composition of oral bacterial communities [60,61]. In line with this hypothesis, a study of 161 subjects observed a large baseline microbiota in more than 98% of the subjects tested, with no differences in relation to diet type (omnivorous, ovo-lacto-vegetarian, or vegan) [62]. In contrast, another study observed small differences in salivary metabolomic profiles in relation to the diet practiced: omnivorous, ovo-lacto-vegetarian, or vegetarians. This would be due to the influence that diet has on the gut microbiota, which in turn influences the oral microbiota [63].

## 5. Impact of Macronutrients during Periodontitis

Macronutrients are the main foods that need to be consumed in large quantities, as they are the most important source of energy for the human organism. These include carbohydrates (or carbohydrates), fats (more correctly, lipids), and proteins [64].

### 5.1. Carbohydrates

Carbohydrates are an essential source of energy for the body and are involved in fat metabolism [65]. Complex, low-glycemic, non-processed, and typically fiber-rich carbohydrates (such as fruits, whole grains, vegetables, and legumes) are usually healthy, while fermentable, high-glycemic, processed, and usually fiber-poor carbohydrates (such as refined sugar, white wheat flour, and sugar-sweetened beverages) have the potential to contribute to chronic inflammation [66,67,68]. Therefore, high intake of high-calorie carbohydrates induces systemic and oral pro-inflammatory actions [69]. The Food and Drug Administration in United States recommended a reduced intake of added sugars and refined grains [70]. Observing these recommendations may reduce the risk of diabetes, weight gain, dental caries, and several oral diseases. The limitation of the intake of refined carbohydrates is also necessary to guarantee an adequate intake of essential nutrients and dietary fiber [8]. A 2014 study indicated that refined sugar intake among adults in the United States has risen by more than 30% over the past three decennia [71]. Recent evidence suggests that consumption of high-glycemic foods alone might increase gingival and periodontal inflammation and bleeding as well, while a diet abundant in complex carbohydrates, with no increase in total energy intake, may lower the risk of gingivitis and periodontitis [72,73,74,75,76,77]. A seminary investigation has indicated that refined carbohydrates are a risk factor for both caries and periodontal disease. [78].

The digestion of carbohydrates begins in the oral cavity through the enzyme salivary α-amylase, which catalyzes the hydrolysis of starch by breaking down the α- 1,4-glycosidic bonds to produce small saccharide units, such as maltose, maltotriose, and alpha-limit dextrins [79]. They are the substrate of several micro-organisms of the oral microbiota and some periodontal pathogens [80]. The intake of refined carbohydrates has increased more and more in recent years, promoting the growth of saccharolytic microbes. These microbes have a favorable nutritional environment and are able to become the predominant micro-organisms in the oral microbiota, outcompeting slower-growing species with alternative nutritional requirements [81]. The saccharolytic bacteria include Streptococcus, Actinomyces, and Veillonella, which have the unique ability to degrade carbohydrates via the Embden−Meyerhof−Pernas pathway, producing acidic by-products including lactate, acetate, ethanol, and formate [1,82]. These bacteria can cause demineralization of dental enamel and development of carious lesions, and on the other hand, promote bacterial acidogenicity and acidification by increasing cell membrane permeability to protons, induction of H+- ATPase activity, and stimulation of metabolic pathways involved in acid neutralization and alkalinization [83]. The pathways in which sugar is metabolized are also shared between some periodontal pathogens, such as Fusobacterium and Prevotella, which can similarly cause acidification [1,83]. In one study, it was seen that although glucose is an essential nutrient for the survival of oral bacteria, its exogenous supply can be discontinuous, and the depth of the periodontal pocket can influence its concentration within a periodontal pocket. Glucose concentration is an environmental factor that may or may not favor the virulence of oral bacteria. It has been observed that *Provetella intermedia*, *Prevotella nigrescens*, and *Porphyromonas* increased their growth in the presence of glucose; at the same time, they significantly reduced the production of cytotoxic end products (succinate, isobutyrate, isovalerate, and ammonia) and proteolytic activities (immunoglobulin, albumin, and casein degradation activities). In contrast, the activity of the main periodontopathic bacterium P gingivalis is not affected in any way by the presence of glucose [84]. However, the degradation of protein substrates plays an important role in the pathogenesis of the periodontal disease [83].

### 5.2. Proteins

The action of proteins in systemic inflammation is still unclear, although many studies suggest that they have a neutral role [85]. In the last years, it has been observed how proteins’ biological activities and their role in genetic inflammation are related to their origin. Currently, “genetic inflammation” is defined as the action that various environmental stimuli, including diet and the oral or intestinal microbiota, can have in altering DNA methylation and histone signatures, altering gene expression, and promoting the development of various local and systemic diseases. The influence of the microbiota on the development of various diseases, oral and systemic, is determined by its ability to influence the release of inflammatory cytokines and the production of antimicrobial peptides. They influence the epigenome through short-chain fatty acid synthesis, vitamin synthesis, and nutrient absorption [86].

Animal protein has been indicated to increase insulin-like growth factor 1, which is implicated in carcinogenesis [70,87]. There are few studies which have investigated the role of protein in periodontal disease. Instead, vegetable protein has been linked to a decreased risk of cardiovascular disease, type 2 diabetes mellitus, and kidney disease [88]. In accordance with the findings, several European guidelines have recommended decreasing meat intake to less than 500 g per week, especially processed meat. A cross-sectional clinical study by Staufenbiel et al. evaluated the periodontal status of 100 vegetarian subjects compared with that of 100 nonvegetarian individuals. The findings indicated that the vegetarian group had significantly less pocket depth, less bleeding on probing, and better oral hygiene when compared with the nonvegetarian group. Therefore, the vegetarian diet appears to affect periodontal health positively. Moreover, the authors pointed out that the vegetarian patients presented a higher level of education and healthier lifestyle than the non-vegetarians [89].

During the development of periodontal disease, the stresses of a protein-rich, neutral-alkaline environment promote the growth of periodontal pathogens [90]. The increased depth of the gingival pocket and the increased production of gingival crevicular fluid induces an increase in bacteria involved in protein degradation. They are broken down into peptides and amino acids (aspartate, serine, and cysteine) through the action of host proteases and peptidases [83]. Amino acids are fermented to generate short-chain fatty acids, such as propionate, butyrate, succinate, acetate, and formate [83]. Fermentation of amino acids neutralizes acidic environments, creating a more favorable atmosphere for the growth of other periodontitis’ pathogens [83]. Some bacteria, including *Prevotella intermedia*, exist as both proteolytic and saccharolytic, depending on environmental pressures [84].

### 5.3. Lipids

Besides being an excellent source of energy, Lipids constitute important structural and metabolic components (such as cell membranes and hormones). Much research has indicated that saturated fatty acids (trans fats and omega-6 fatty acids) are unhealthy, as they are implicated in promoting inflammatory conditions [85]. These are commonly found in the following foods: industrial meat, dairy products, eggs, and vegetable oils (safflower oil, sunflower oil, grape seed oil, and margarine). Trans fatty acids are the result of overheating fats and are therefore typical of particular forms of cooking foods, such as frying, baking, or roasting [91]. In a longitudinal study conducted with 264 Japanese participants, a statistically positive association between saturated fatty acids and the occurrence of periodontal lesions was indicated [92]. In contrast, studies on omega-3 fatty acids have been analyzed extensively over the past several years, as they have been shown to be associated with reduced and systemic inflammation [85]. The influence of dietary lipids on the oral microbiota remains unknown.

Table 1 resumes the association between macronutrients and their effects in periodontal tissues.

## 6. Impact of Micronutrients during Periodontitis

“Micronutrients” include vitamins, minerals, and trace elements. They are essential for proper functioning of the body in amounts less than 100 mg/day. They have been observed to be involved in periodontal disease. [93]. Vitamins A, D, E, and K have the characteristic of being fat-soluble and dissolve in fat, whereas vitamin B complex vitamins and vitamin C are hydrophilic and dissolve in water. Micronutrient deficiency can be associated with several conditions: taking certain medications: (antacids, antibiotics, antihypertensives, chelating agents, corticosteroids, diuretics, laxatives, NSAIDs), malabsorption syndrome or diarrhea; lifestyle factors (diet, malnutrition, chronic alcohol or nicotine abuse, and consumption of fast food or processed foods); systemic disorders (diabetes mellitus, thyroid and parathyroid disorders); increased requirements (pregnancy, lactation, growth, physical/mental stress); and other physiological and nutritional factors that may vary throughout life. Bioavailability is often associated with the chemical form and co-presence of other micronutrients, such as vitamin D and calcium [94]. Positive effects on periodontal health have been associated a several vitamins [95,96].

### 6.1. Vitamin A

Vitamin A is a liposoluble vitamin involved in maintaining the integrity of epithelial cells [97]. Vitamin A is taken through the diet, and foods that are rich in it include: eggs, cod oil, carrots, peppers, liver, sweet potatoes, broccoli, and leafy greens. Vitamin A deficiency leads to retinal disorders (such as night blindness and hyperkeratosis). Vitamin A has a potential antioxidant effect, for which it has been incorporated into non-surgical and surgical periodontal treatment [38]. High dietary intake of beta-carotene (≥7.07 mg/d) was associated by Dodington et al. with a significantly lower percentage of sites with probing depth >3 mm after nonsurgical periodontal treatment. These effects on periodontal parameters were found to be greater in adult nonsmokers than in adult smokers with chronic periodontitis [8]. However, the role of vitamin A in periodontal therapy needs further investigation because there are few studies present. This can probably be attributed to the hepatic toxicity it may induce in excess. [39].

### 6.2. Vitamin B

The vitamin B complex family includes vitamins B1 (thiamine), B2 (riboflavin), B3 (niacin), B5 (pantothenic acid), B6 (pyridoxine, pyridoxine, pyridoxamine), B7 (biotin), B9 (folic acid), and B12 (cobalamins) [98]. They are implicated in several processes: metabolism, muscle development, erythrocyte production, and collagen synthesis [99]. Dietary sources of vitamin B12 include meat (beef, pork, offal), poultry, eggs, fortified cereals, milk and milk products (dairy), and seafood (clams, oysters), mackerel, and salmon [98]. Deficiency induces weakness, fatigue, psychosis, megaloblastic anemia, dermatitis, cheilitis, tongue glossitis, white matter damage in the spinal cord and brain, irritability, and peripheral neuropathy [100]. Low serum levels of vitamin B12 have been associated with an increased risk of developing periodontal lesions [95], explaining the importance of vitamin B12 supplementation for vegans, and sometimes for vegetarians as well [96]. In a double-blind, placebo-controlled study, the activity of a folate-containing mouthwash in patients with gingivitis and periodontitis was evaluated. From the results, it was observed that the use of the aforementioned mouthwash for 4 weeks induced a reduction in probing bleeding and gingival redness compared with patients who used placebo for the same period [101].

### 6.3. Vitamin C

Vitamin C (ascorbic acid) is a water-soluble vitamin, which is contained in many vegetables and fruits. It is an effective antioxidant involved in collagen synthesis, tyrosine metabolism, and noradrenaline synthesis [102]. Vitamin C deficiency causes scurvy. All these functions and activities have given vitamin C an interesting potential role in periodontal health [103]. Several cross-sectional studies have indicated low serum vitamin C values and lower vitamin C intake in subjects with periodontitis compared with controls [39,104]. Several cross-sectional studies have indicated low serum vitamin C values and lower vitamin C intake in subjects with periodontitis compared with controls [39,104]. In agreement with this, two clinical studies reported that increased ingestion of fruits containing vitamin C (such as grapefruits, peppers, kiwis, etc.) could decrease gingival and periodontal inflammation [73,105]. Therefore, regardless of oral hygiene, vitamin C reduction induces gingival bleeding [106,107].

### 6.4. Vitamin D

Vitamin D includes all forms of the hormone cholecalciferol, the biologically active form of which is 1,25-dihydroxycholecalciferol (1,25(OH)2D3) [108]. It is implicated in the regulation of calcium-phosphate homeostasis and bone mineral metabolism [109]. Furthermore, vitamin D increases intestinal calcium absorption by decreasing the parathyroid hormone secretion, consequently decreasing systemic bone resorption [110]. Vitamin D also stimulates osteoblastic bone production and alkaline phosphatase activity, promoting bone remodeling [111,112]. It is widely noted how essential vitamin D is in calcium absorption and bone metabolism regulation. While still little is known on this topic, it is widely accepted that vitamin D plays an active role in regulating the immune system, performing innumerable anti-inflammatory activities [113,114]. Laky et al., through a case-control study, observed that patients with periodontal disease were common with higher vitamin D deficiency (<50 nmol/L) than healthy subjects [115]. Vitamin D values were found to be inversely related to clinical attachment loss and tooth loss in two cross-sectional studies [115,116]. However, some studies have reported values that counteract this association, making further randomized, controlled interventional studies necessary [93].

### 6.5. Vitamin E

Vitamin E is the most important fat-soluble antioxidant, performing proactive activity against lipid peroxidation of polyunsaturated fatty acids in cell membranes [117,118]. It reduces the activity of cyclooxygenase, lipoxygenase, and phospholipase A2 (reducing the synthesis of prostaglandin E2, leukotriene B4, and thromboxane A2) and inhibits the activity of protein kinase C (reducing leukocyte adhesion and the formation of reactive oxygen species) and the formation of tumor necrosis factor alpha (TNFα). Vitamin E is found in vegetable oils, unprocessed cereals, and nuts. To a lesser extent, we find it in fruits, vegetables, and meat (especially the fatty part) [119]. Subjects with periodontal disease show a reduction in vitamin E when compared with healthy subjects [120]. Through their prospective study of 264 patients in Niigata, Iwasaki et al. observed that higher vitamin E intake was inversely associated with the number of teeth with periodontal disease progression. The duration of the study was 2 years.

### 6.6. Vitamin K

Vitamin K includes a group of vitamins necessary for the synthesis of proteins required to form blood clotting factors such as prothrombin and factors VII, IX, and X [121].

Vitamin K has recently been found to be involved in the generation of proteins necessary for bone metabolism, osteocalcin, and periostin [121]. However, the study by Aral et al. indicated that vitamin K supplementation is unable to reduce pro-inflammatory factors in periodontal tissue [122].

Table 2 resumes major vitamins dietary sources and their effects in periodontal tissues.

Currently, there is no direct evidence in the literature of interactions between micronutrient intake and the oral microbiome. It is well established that some bacteria in the gut microbiota, lactic acid bacteria (LAB) and bifidobacteria, can produce vitamins, including vitamin B12. However, in the oral cavity, at present, there are no such observations [123]. In contrast, an association between intake of refined sugars and vegetable oils and micronutrients has been observed: these contain very low concentrations of micronutrients. Therefore, the association between high intake of such foods and increased expression of pathogenic bacterial species in the oral cavity could be partly due to vitamin deficiencies. It is worth mentioning how vitamin deficiency is associated with several oral diseases, such as recurrent aphthous tongue [124].

## 7. Strategies against Dysbiotic Microbiota

In recent decades, probiotics and prebiotics have been widely studied and used for their health benefits, as they are able to maintain the balance of the human microbiota. Probiotics have been described as “live micro-organisms which, when administered in adequate quantities, confer a health benefit on the host” [137]. The healthy activity of probiotics is attributed to several mechanisms: secretion of antimicrobial compounds (lactic and acetic acid, hydrogen peroxide, bacteriocins); production of biosurfactants that limit adhesion to the epithelial surface; degradation of toxins; reduction in LDL cholesterol; stimulation of the host immune system and vitamin synthesis. The probiotics mechanisms of action in the oral cavity are illustrated in Figure 3. Instead, the concept of prebiotics, introduced by Gibson and Roberfroid in 1995, has undergone several modifications. Currently, prebiotics are understood as “substrates that are selectively utilized by host microorganisms that confer a health benefit”. Therefore, prebiotics are nutrients for probiotic bacteria that have positive repercussions for human health. They are mostly undigested fibers, consisting of complex carbohydrates (galactooligosaccharides and inulin), which are not absorbed directly by the body but metabolized by the microbiota. Metabolized prebiotic compounds have several positive effects on the body such as reducing inflammation and modulating the immune system [137].

At present, the pharmaceutical and food industries are promoting two strategies to improve consumer health by exploiting the effects of diet on human health: the addition of probiotics (viable bacteria and yeasts) and prebiotics (inulin, FOS, and others). Probiotics and prebiotics are used in combination because of their symbiotic association. The use of probiotics containing *L. rhamnosus* SP1 was evaluated in addition to non-surgical therapy of chronic periodontitis. Twenty-eight patients were included in the study, who underwent non-surgical therapy, including scaling and root planing, and were randomly assigned to a test (probiotic sachet) or control (placebo) group. The probiotic sachet (*L. rhamnosus* SP1, 2 × 10^7^ CFU) was taken once daily for 3 months. However, the results showed that oral administration of L. rhamnosus SP1 resulted in clinical improvements similar to those in patients treated with scaling and root smoothing alone [138]. In a randomized double-blind study, the benefits of oral administration of heat-inactivated *L. plantarum* L-137 (HK L-137) in relation to the outcome of periodontal therapy were evaluated. The test group received one capsule of HK L-137 (10 mg), while the control group received one capsule of placebo daily for 12 weeks. The results of the study indicate that daily intake of HK L-137 can reduce periodontal pocket depth in patients receiving supportive periodontal therapy [139,140]. In another double-blind, randomized clinical trial involving 40 patients with generalized gingivitis, the efficacy of probiotic products containing bacilli was evaluated. After undergoing professional hygiene sessions, the test group (20 patients) received a coded package containing one toothbrush, one probiotic toothpaste, two probiotic mouthwashes, and one probiotic toothbrush cleaner for 8 weeks, while the control group received identical placebo products. The probiotic products contained 5 × 10^7^ CFU of *Bacillus subtilis*, *Bacillus megateriume* spores of *Bacillu pumulus*. Plaque and gingivitis indices were significantly reduced after 8 weeks of treatment but showed no statistically significant differences between the placebo and probiotic groups [141]. Currently, probiotics are also used in the treatment of various pathological conditions of the oral cavity that underlie a dysbiosis of the oral microbiota. The effects of probiotics on oral health are illustrated in Table 3.

**Figure 4:**
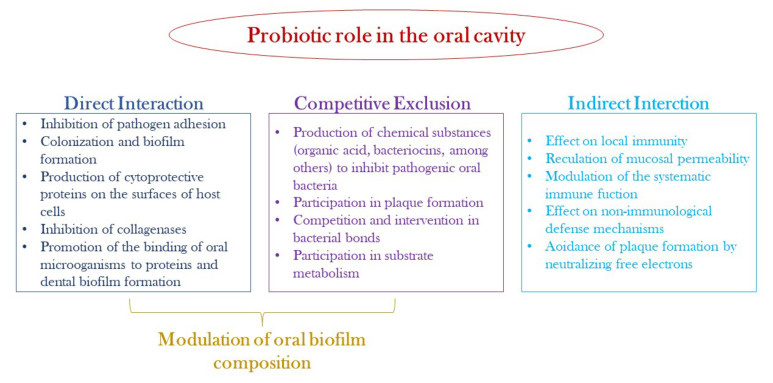
Possible mechanisms of probiotic action in the oral cavity.

**Figure 5:**
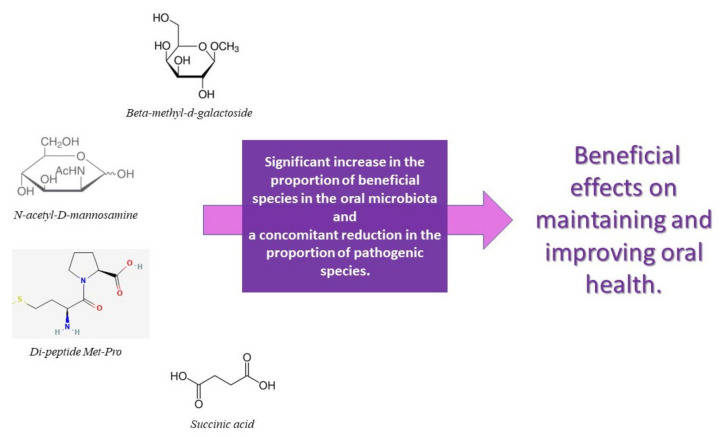
List and action of the main prebiotics that confer oral health benefits.

Several studies investigate the role of probiotics on periodontal health, while there is a lack of interventional studies on the effects of prebiotics on periodontal disease [151]. Selective stimulation of native beneficial bacteria can be induced by increasing their growth or by increasing their metabolic activity, thereby increasing their competition with pathogens. A first attempt has been made to exploit this concept in the oral cavity. In a recent study, Slomka et al. identified two prebiotics, beta-methyl-d-galactoside and N-acetyl-d-mannosamine, which could be used in the promotion of bacteria associated with oral health [76]. A recent in vitro study showed that treatment of multi-species biofilms with N-acetyl-D-mannosamine, succinic acid, and the di-peptide Met-Pro resulted in a significant increase in the proportion of beneficial species in the oral microbiota and a concomitant reduction in the proportion of pathogenic species. It is important to specify that the efficacy of N-acetyl-D-mannosamine in a nutrient-rich environment, recreated similar to that of the oral cavity, is not reduced. N-acetyl-D-mannosamine, succinic acid, and the di-peptide Met-Pro are promising oral prebiotic compounds, which have beneficial effects on maintaining and improving oral health. However, for them to be defined as true oral prebiotics, their action needs to be demonstrated in in vivo studies, taking into account even more complex interbacterial interactions and fluctuations in environmental factors [152]. Figure 4 and Figure 5 schematizes how the main prebiotics influence oral health, particularly periodontal health.

## 8. Conclusions

The evidence of the abovementioned studies has shown that diet plays an important role in the typical oral dysbiosis of periodontal disease, as it provides nutritional sub-strates for micro-organisms, can promote the creation of a microenvironment suitable for the multiplication and survival of certain periodontal pathogens bacteria, and can inhibit the growth of other micro-organisms. Among foods, with the greatest impact on periodontal disease, we find proteins and simple carbohydrates, which on the one hand favor the establishment of a microenvironment with an acid pH and, on the other, favor inflammation. In conclusion, to promote periodontal health, reduce inflammation, and promote eubiosis, we believe that multiple benefits are induced, in addition to mechanical removal of biofilm through non-surgical periodontal therapy, by dietary control and the associated intake of vitamin supplements and probiotics, prebiotics, or symbiotic agents.

## Figures and Tables

**Table 1:** Association between macronutrients and periodontal health.

Nutrient	Dietary Sources(s)	Importance in Oral and Periodontal Health	Reference
**Proteins**	Proteins of vegetable origin (pulses, some vegetables, cereals) Proteins of animal origin (meat, fish, milk, dairy products, cheese, eggs)	During the development of periodontal disease, the stresses of a protein-rich, neutral-alkaline environment promote the growth of periodontal pathogens, with a worsening of periodontal clinical parameters.	[88,90]
**Flats (lipids)**	Saturated fatty acids (butter, palm oil, cheese) Monounsaturated fatty acids (olive oil) Omega-6 polyunsaturated fatty acids: of vegetable origin (grape seed oil, soya oil, sunflower oil, nuts) and of animal origin (lard, chicken egg yolk, in poultry, fish) Omega-3 polyunsaturated fatty acids: of vegetable origin (salmon, mackerel, herring, sardines, cod liver oil)	There is a statistically positive association between saturated fatty acids and the occurrence of periodontal lesions. On the contrary, omega-3 fatty acids have been studied intensively in recent years because they are associated with less systemic and oral inflammation. Several studies have observed that omega-3 fatty acids, in addition to periodontal therapy, have shown significant benefits in terms of reducing pocket depth and increasing attachment.	[85,91,92]
**Carbohydrates**	Low-glycemic (fruits, whole grain, vegetables, legumes) High-glycemic (refined sugar, white wheat flour, sugary drinks)	Consumption of high-glycemic foods may increase gingival and periodontal inflammation and bleeding; in contrast, a diet rich in complex carbohydrates may reduce the risk of gingivitis and periodontitis. High intakes of processed carbohydrates are a risk factor for the development of caries.	[65,65,66,67,68,69, 71,72,73,74,75,76,77,78].

**Table 2:** Association between deficiencies of certain micronutrients and periodontal health.

Nutrient	Dietary Sources(s)	Importance in Oral and Periodontal Health	Reported Improvement in PD and CAL (Mean mm, SD)	References
**Vitamin A**	Cod liver oil, carrots, capsicum, liver, sweet potato, broccoli, leafy vegetables	Not clear. Research indicates insignificant improvement in periodontal health upon supplementation.	PD: 0.52 ± 0.03 CAL: n.d.	[125,126]
**B-vitamins**	B1—Liver, oats, pork, potatoes, eggs B2—Bananas, dairy, green beans B3—Eggs, fish, meat, mushrooms, nuts B5—Avocados, meat, broccoli B6—Meat, vegetables, nuts, banana B7—Raw egg, liver, leafy vegetables, peanuts B9—Cereals, leafy vegetables B12—Animal products	Supplementation may accelerate post-surgical healing and improve CAL and BOP.	PD: 1.57 ± 0.34 CAL: 0.41 ±0.12	[99,101]
**Vitamin C**	Citrus fruits, vegetables, grapefruits, peppers, kiwis, liver	Gingival bleeding and inflammation are hallmarks of scurvy. Supplementation may reduce gingival and periodontal inflammation and may improve outcomes of periodontal therapy.	PD: 0.58 ± 0.14 CAL: n.d.	[66,105,127]
**Vitamin D**	Fish eggs, mushrooms, liver, milk	Deficiency may lead to delayed post-surgical healing. Supplementation may reduce BOP and alveolar bone loss. Local application may accelerate post-surgical healing/osseointegration.	PD: 1.35 (SD n.d.) CAL: 1.4 (SD n.d.)	[97,99,128,129,130]
**Vitamin E**	Green vegetables, egg yolk, vegetable oils, unprocessed cereals, nuts	Deficiency may lead to gingival bleeding. Supplementation may reduce periodontal inflammation and hinder periodontitis progression. No known effects on periodontal therapy if supplementation used as an adjunct.	PD: 0.39 ± 0.18 CAL: n.d.	[117,119,125,131,132,133,134]
**Vitamin K**	Green vegetables, egg yolk, kale, spinach, cabbage, mustard	Deficiency may lead to gingival bleeding. Supplementation seems to be unable to reduce periodontal inflammation.	n.d.	[121,122,135,136]

PD (pocket depth), CAL (clinical attachment level), BOP (bleeding on probing), n.d. (not determined), SD (standard deviations).

**Table 3:** Effects of probiotics in the oral cavity.

Oral Diseases	Probiotic Strain	Patients	Vehicle	Results	References
**Halitosis**	*L. reuteri* -DSM 17938 -ATCC PTA 5289 *L. salivarius* WB21 *L. salivarius, L. reuteri*	V: 25 healthy adults; Mean age: 22 years V: 23 healthy adults; Age: 22–67 years V: 32 healthy adults; Age: 25–59 years	Chewing gum Tablets Capsules	Probiotic chewing gum had beneficial effect on oral malodor in organoleptic score but not on levels of volatile sulfur compounds. Probiotic tablets help to control oral malodors and malodor-related factors. Probiotic capsule consumption significantly reduced plaque and modified gingival bleeding indices at 3 months.	[142] [143] [144]
**Dental caries**	*L. reuteri* -DSM 17938 -ATCC PTA 5289 *L. reuteri* ATCC 55730 *L. rhamnosus* SP1	V: 36 healthy youth; Age: 12–17 years V: 113 children; Age: From birth up to 1 year of age V: 261 children; Age: 2–3 years	Tablets Oil drops Milk	Probiotic tablets showed a beneficial tendency on early, non-cavitated caries lesions in adolescents. Daily probiotic consumption was associated with reduced caries prevalence and gingivitis score in the primary dentition at 9 years of age. The intake of milk supplemented with probiotics decreased the prevalence of caries and the development of cavitated lesions.	[145] [146] [147]
**Periodontitis**	*L. rhamnosus* SP1 *L. plantarum* L-137 *L. rhamnosus* GG, B. lactis BB-12	V: 28 adults; Mean age: test group 52.7 ± 7.3 years; control group 46.9 ± 10.3 years V: 39 patients; Mean age: 66.2 years V: 108 healthy adolescents; Age: 13–15 years	Sachet Capsules Lozenges	Oral administration of probiotic sachet resulted in clinical improvements similar to scaling and root planing. Daily consumption of heat-inactivated L. plantarum L-137 decreased the depth of periodontal pockets. Intake of lozenge probiotics improved gingival health and decreased the presence of harmful bacteria.	[148] [149] [150]

V = volunteers; L. reuteri = Lactobacillus Reuteri; L. salivarius = Lactobacillus salivarius; L. rhamnosus = Lactobacillus rhamnosus; L. plantarum = Lactobacillus Plantarum.

## Data Availability

Data are available from the corresponding author upon reasonable request.
